# Selenium, Zinc, and Manganese Status in Pregnant Women and Its Relation to Maternal and Child Complications

**DOI:** 10.3390/nu12030725

**Published:** 2020-03-10

**Authors:** Sehar Iqbal, Inayat Ali, Petra Rust, Michael Kundi, Cem Ekmekcioglu

**Affiliations:** 1 Department of Environmental Health, Center for Public Health, Medical University of Vienna, Kinderspitalgasse 15, 1090 Vienna, Austria; saheriqbal55@gmail.com (S.I.); michael.kundi@meduniwien.ac.at (M.K.); 2 Department of Social and Cultural Anthropology, University of Vienna, Universitätsstrasse 7, 1010 Vienna, Austria; inayat_qau@yahoo.com; 3 Department of Nutritional Sciences, University of Vienna, Althanstrasse 14, 1090 Vienna, Austria; petra.rust@univie.ac.at

**Keywords:** selenium, zinc, manganese, pregnancy, maternal complications, child complications

## Abstract

Micronutrients, as essential components of prenatal care, are important to reduce the risk for maternal and child morbidity and mortality by lowering pregnancy-related complications. The present study aimed to investigate the status of the trace elements, i.e., selenium, zinc, and manganese in pregnant and non-pregnant women from a developing country and to evaluate its relationship with maternal and child complications. Selenium, zinc, and manganese concentrations were measured in the blood serum of 80 pregnant women and compared with 40 non-pregnant healthy controls. The quantitative analyses of trace elements were performed by using the inductively coupled plasma–optical emission spectrometry (ICP-OES) method. The information about the dietary habits of the study participants was recorded by using a food frequency questionnaire. The results showed significant lower selenium and zinc levels in pregnant women as compared to the controls (2.26 ± 1.09 vs. 2.76 ± 1.15 µmol/L, *p* = 0.031; 21.86 ± 7.21 vs. 29.54 ± 7.62 µmol/L, *p* < 0.001) respectively, with no difference in manganese concentrations (1.40 ± 0.09 vs.1.38 ± 0.09 log_10_ nmol/L, *p* = 0.365). Regarding maternal and child complications, higher manganese levels were associated with an increased odds ratio for maternal complications (OR = 3.175, CI (95%) 1.631−6.181; *p* = 0.038). Consumption of dairy products was associated with lower selenium and manganese values. Pregnant women showed a lower serum selenium and zinc status, and in addition elevated serum manganese concentrations, which might be associated with a higher risk for maternal pregnancy/birth complications, although more studies are necessary to evaluate this association.

## 1. Introduction

Micronutrients, including trace elements, are virtually involved in all biological and metabolic activities, including tissue growth, cell signalling, motility, proliferation, and apoptosis [1]. As a vital component of prenatal care, these elements help to reduce the risk for maternal and child morbidity and mortality by lowering pregnancy-related complications [2]. An adequate supply of trace elements is essential for healthy fetoplacental development and to repair any cellular damage during pregnancy [3]. For instance, selenium is vital for efficient antioxidant defence in both mother and foetus [4]. In particular, as an essential functional component of the antioxidative enzyme glutathione peroxidase, selenium helps to prevent adverse foeto-maternal outcomes such as miscarriages, neural tube defects, preeclampsia, premature rupture of membranes, and gestational diabetes [5]. On the contrary, weak placental antioxidant defence due to low maternal plasma selenium concentration might increase the risk of small for gestational-age infants [6].

In addition to selenium, adequate zinc intake has been shown to be a decisive factor for successful embryogenesis and to reduce the risk of preterm births [7]. Apart from this, zinc, as an essential trace element, plays a key role in protein synthesis, cell signalling, oxidative defence, growth, and tissue maintenance [8]. Homeostatic changes during the gestational period may result in increased zinc utilisation and mitigate immediate detrimental effects as a result of zinc deficiency [9]. The findings of a recent systematic review showed an association of low maternal dietary zinc intake with an increase in pregnancy complications [10]. In agreement, maternal zinc deficiency intensifies the danger of low birth weight and small for gestational age infants [11].

Manganese is another essential trace metal that is found in almost all tissues and is needed for reproduction, digestion, regulation of blood sugar, and haemostasis [12]. It is one amongst the most ubiquitous trace elements knowingly involved in bone growth, immune system, cellular energy, and in the healthy metabolism of lipids, proteins, and carbohydrates [13]. Being a constituent of manganese catalase and Mn-superoxide dismutase (SOD), manganese helps to minimise oxidative stress through detoxification of superoxide-derived free radicals [14]. Although manganese is essential for different enzymatic functions and homeostatic mechanisms, the excessive bodily accumulation of this element can result in toxicity and neurologic impairment [15].

During gestation, manganese is considered an essential mineral nutrient needed for proper foetal development [16]. However, also an inverted U-shaped relation has been found between maternal blood manganese and infant motor and cognitive functions [17]. Apparently, both, high as well as low, manganese levels have an association with adverse birth outcomes [18]. An increase in manganese exposure has been implicated as a potential risk factor for gestational hypertension [19]. In contrast, low maternal manganese concentration during the third trimester may augment the risk of low birth weight infants [20].

Diet and physiological changes in digestion, absorption, and utilisation influence the availability of trace elements during pregnancy. Moreover, the imbalance of all the trace elements mentioned above has been closely related to perinatal complications and poor pregnancy outcomes [21]. However, owing to differences between populations, lifestyle, diet, and living environments, changes of these trace elements in Pakistani pregnant women have not been studied so far. As a low-income country, Pakistan has reported widespread micronutrient deficiencies among its most vulnerable population groups, such as children and women of reproductive age [22]. For example, in a previous paper, we showed that Pakistani pregnant women have lower concentrations of the iron storage marker ferritin and urinary iodine levels compared to controls [23].

To our knowledge and through a literature search, there is almost no information available regarding the trace element status and its relation to pregnancy outcomes in women from Pakistan. Moreover, very few studies have combined the analyses of selected trace elements in one population. Therefore, considering both the potential public health burden of malnutrition and a dearth of the available data in the country, we evaluated the selenium, zinc, and manganese status in Pakistani women.

The objectives of this study were to evaluate maternal serum selenium, zinc, and manganese status during pregnancy, comparing these levels to non-pregnant healthy controls, and analysing the association of these trace elements with maternal and child complications. The results would improve the evidence regarding the importance of an adequate status of trace elements during pregnancy in a developing country.

## 2. Materials and Methods

### 2.1. Study Design and Sample Population

This cross-sectional comparative study was conducted to analyse the status of selected trace elements in pregnant and non-pregnant women aged 26 ± 4 and 25 ± 4 years respectively. Characteristics of study participants are shown in Table 1. A total of 80 pregnant women during their third trimester and residing in the same region were recruited from the Obstetrics and Gynaecology Department, District Headquarter Hospital Khanewal, Pakistan. Another 40 age-matched, healthy, non-pregnant women who were registered to the same hospital for the routine obstetrical examination were requested to participate as a control group. All the participants signed an informed written consent form in the local language (Urdu), and the study was approved by the National Bioethics Committee Pakistan (reference no. 4-87/NBC-281/17/1439) and is in accordance with the tenets of the Helsinki Declaration.

### 2.2. Sociodemographic Data and Food Frequency Questionnaire

Several variables, including socio-demographic and lifestyle factors, may confound the relationship between pregnancy outcomes and foeto-maternal micronutrient deficiencies. Therefore, a predesigned questionnaire was used to collect personal and sociodemographic data, such as age; residence; educational level; family size; income; socioeconomic status; and lifestyle, for instance, smoking (no participant smoked), physical activity, or being on any specific diet (no participant was on any diet). The target of ≥20 minutes of exercise on most days/week was used to assess the physical activity level of the study population [24]. Body mass index (BMI) was measured at the time of delivery and obstetric data, such as gravidity, parity, antenatal visits, vaccination status, and obstetric complications in previous pregnancies or deliveries were also recorded. Women who were taking vitamin and mineral supplements were excluded, although a total of five women reportedly were taking folic acid supplements during their pregnancies.

In order to obtain information about the dietary habits of the selected women, dietary intake of both study groups was evaluated by using a 17-item food frequency questionnaire (FFQ). The food frequency questionnaire was based on a previous study by Haftenberger et al. [25]. The FFQ was comprised of food intake data (daily, weekly, monthly, and never) concerning consumption of meat, fish, processed food, dairy products, eggs, grains, rice or noodles, legumes and pulses, vegetables, fruits, salad, oils, nuts, sweets, snacks, and cold and hot drinks.

### 2.3. Maternal and Child Complications

The information regarding date and mode of delivery; infant’s sex; parity; and infant anthropometrics, such as birth weight, appearance (skin colour), pulse (heart rate), grimace response (reflexes), activity (muscle tone), and respiration were recorded by two instructed nurses of the selected hospital. From the last five parameters, the Appearance, Pulse, Grimace, Activity, Respiration (APGAR) score was calculated on the basis of criterion scales from 0 to 2, resulting in scores ranging from 0 to 10. For this purpose, scores of 7 and above were considered as normal; 4 to 6, fairly low; and 3 and below, critically low.

Moreover, data related to prenatal mortality, duration of gestation, miscarriages, premature rupture of membranes, intrauterine growth restriction, small for gestational age, preterm birth, and placental weight were also recorded. Low birth weight was defined as a birth weight less than 2500 g, regardless of gestational age, whereas small for gestational age infants were defined as those with birth weights below the 10th percentile for their gestational age, as established by the World Health Organisation (WHO) [26].

### 2.4. Analyses of Trace Elements

The blood specimens of pregnant women, at the time of delivery and 24 hours before delivery in the case of a recommended caesarean delivery, were collected by the assigned trained nurses of the district hospital Khanewal. Similarly, the blood samples of 40 age-matched healthy women of reproductive age who participated as a control group was taken by well-trained hospital staff for analyses. Samples were taken in gel vials, centrifuged at 3000 rpm for 10 minutes to separate serum, and serum samples were stored at −20 °C until analyses.

Serum concentrations of selenium, zinc, and manganese were analysed by direct aspiration of liquid samples into an inductively coupled plasma–optical emission spectrometry (ICP-OES) system and quantified against certified reference material (Multi elements, Merck, Burlington Massachusetts, USA).

Different concentration standards were prepared, ranging from 0.1 µg/L to 500 µg/L and aspirated into the ICP-OES system before conducting sample analysis. All trace elements had good linear concentration–response curves with a correlation coefficient >0.999. The polychromator was purged with a low flow of either argon or nitrogen for the detection of emission lines of low UV wavelengths. ICP Expert II software version 1.0 was used for instrument operations. Three replicate readings were taken with a read time of 30 seconds.

### 2.5. Statistical Methods

For sample size determination, a prerequisite to detect an effect size of Cohen’s *d* = 0.7 at the overall significance level of 5% with a power of 80% and a ratio of 2:1 for pregnant women to controls was implemented. Therefore, the required sample sizes were established as 77 and 39 with rounded figures to 80 and 40, which was appropriate to detect an odds ratio of 2.5 for maternal and child complications. Unpaired *t*-tests or chi-squared tests were applied to evaluate the statistical differences of the study groups.

A preliminary analysis of variance, including the primary independent variables together with the covariates, was performed for all trace elements. The residuals of these analyses were tested for normality by applying Kolmogorov–Smirnov tests with Lilliefors’ corrected *p*-values. Logarithmic transformation was necessary for manganese due to significant deviations from normality. General linear model analyses controlled for age, BMI, education, income, physical activity, and parity were performed to evaluate the trace element status between pregnant women and the control group.

Because multiple pregnancies result in a higher maternal nutrient drain and an accelerated depletion of nutritional reserves [27], subgroup analysis with comparisons between primi- and multipara was executed and additionally controlled for antenatal visits and previous miscarriages.

Binary logistic regression analyses were performed to investigate the association of the trace element status with maternal and child complications. For this purpose, all maternal complications or all child complications were included as the dependent variable (yes/no) into the model, whereas trace element concentrations (selenium, zinc, and manganese) were used as the independent variable. Age, parity, income status, education levels, antenatal visits, and physical activity were taken as covariates, and data were presented as odds ratios with 95% confidence intervals.

A stepwise multiple regression analysis was carried out to analyse the impact of food intake on trace element status in both pregnant and non-pregnant women. In this regard, data from the FFQ were standardized to weekly intake data. Data were controlled for age, BMI, income, physical activity, and marital status.

All statistical analyses were performed by SPSS, version 25 (IBM Corp., Armonk, NY, USA). A *p*-value of <0.05 was considered significant.

## 3. Results

The socio-demographic data showed that 56.3% of pregnant women and 47.5% control women had no education, and that most of the pregnant women were housewives as compared to controls. In addition, 32.5% of the pregnant women registered were with one or more pregnancy complication (maternal and child) and 33.8% reported a history of previous miscarriage (Table 1). The maternal and child complications were premature rupture of membranes (5%), bleeding (8.8%), hypertension (6.3%), small for gestational age (8.8%), stillbirth (5%), preterm birth (5%), low birth weight (12.6%), and low APGAR score (8.8%).

Our results showed that selenium levels were significantly lower in pregnant women as compared to the control group (Table 2). Additionally, lower levels of zinc in pregnant women were found as compared to non-pregnant controls (21.86 ± 7.21 vs. 29.54 ± 7.62 µmol/L, *p* < 0.001), whereas there was no difference of log_10-_ manganese levels between both groups (Table 2).

In a sub-group analysis of pregnant women (primipara vs. multipara), lower levels of log_10-_ manganese was found in multipara pregnant women (1.38 ± 0.10 log_10_ nmol/L) as compared to primipara (1.43 ± 0.10 log_10_ nmol/L, *p* = 0.025), whereas no difference was observed regarding selenium and zinc serum concentrations (Table 3).

### 3.1. Association of Selenium, Zinc, and Manganese Status and Risk of Maternal or Child Complications

The association of selenium, zinc, and manganese status with all maternal and child complications was determined by using binary logistic regression analyses. Higher zinc levels were associated with lower odds ratios of maternal complication, though not reaching statistical significance. On the contrary, higher manganese levels were found to be associated with an increased odds ratio of maternal complications (*p* = 0.038). However, no association was found for child complications (Table 4).

### 3.2. Food Intake and Correlation of Different Foods With the Trace Element Status

Weekly intake of grains (wheat), fruits, eggs, and dairy products was relatively high in both groups as compared to other food groups, and intake of meat, fish, and also sweets and snacks were low. In the group of pregnant women, 91.2% women had an intake of grains one to three times a day and 8.8% consumed grains four or more times per day, whereas all women of the control group had grains intake one to three times a day. Compared to the pregnant women, 30% of women of the control group had meat intake 1–3 times a week and 15% ate meat 4–7 times a week, whereas, in the group of pregnant women, only 10% consumed meat 1–3 times a week, and 7.5% ate meat 4–7 times a week. However, the consumption of dairy products was high in the group of pregnant women (67.5% once/day, and 25.1% more than two times a day) as compared to controls (55% once/day).

Moreover, a stepwise multiple regression analysis was performed to evaluate the correlation between food intake and levels of selenium, zinc, and manganese. Most of the food items were not correlated with the trace element status. However, increased consumption of dairy products was associated with decreased levels of manganese and to a lower extent also of selenium (Table 5). Increased consumption of local and traditional cold drinks such as sherbet, milkshake, buttermilk, and lassi, was associated with increased levels of zinc, whereas increased consumption of grains and sweets were found to increase manganese levels (Table 5).

## 4. Discussion

The objectives of our study were to investigate the status of the trace elements, i.e., selenium, zinc, and manganese in pregnant and non-pregnant women from a developing country and to evaluate its relationship with maternal and child complications.

A major result of our study was that a low concentration of serum selenium and zinc was found in Pakistani pregnant women as compared to a control group. Additionally, in accordance with our findings, some previous studies [28,29] showed a low selenium and zinc status in pregnant women as compared to non-pregnant control women. However, a study by Kassu and colleagues reported no difference in the mean serum zinc and selenium levels during pregnancy [30]. A comparison between the zinc status during the third trimester of pregnancy in the current study with a study performed in China showed that the zinc levels were higher than in the current findings [31]. Furthermore, regarding the selenium status, Kilinc and colleagues showed lower mean serum selenium concentrations in pregnant women from Turkey [32].

Variances in trace element concentrations could result from different factors, including age, gender, body composition, soil, geographical location, food accessibility, cultural practices (geophagia), and genetics [33]. Selenium enters the food chain through plants; therefore, the soil concentration and variation of food sources largely determine the selenium status of an individual [34]. However, the changes in selenium status during pregnancy could be due to plasma volume expansion or also high utilisation of selenium for producing antioxidant compounds such as glutathione peroxidase and selenoprotein-P to prevent oxidative damage [35].

Moreover, the possible reasons for zinc deficiency during pregnancy can be explained, for example, by a decrease in zinc-binding protein levels, or due to the effects of other trace elements [36]. Additionally, a high intake of dietary inhibitors, such as fibre-rich food, and calcium and cereal-based diets (high in phytate), appears to interfere and limit zinc absorption [37]. Besides these, acute maternal stress is another relevant factor to lower zinc levels during pregnancy [32]. The mechanism might be an increased acute phase response during maternal stress, which appears to enhance the synthesis of metallothionein, resulting in a decline in zinc levels [38].

Multiple pregnancies are associated with a higher risk of perinatal complications [39] due to a higher maternal nutrient drain and an accelerated depletion of nutritional reserves [27]. Our observations tend to confirm the mentioned relationship because we found low levels of manganese (and also to some degree selenium) in multigravida women as compared to primipara. In this regard, a previous study found that an increase in maternal parity is associated with lower circulating concentrations of haemoglobin and ferritin levels [40]. Nevertheless, the paucity of literature regarding multiple births and maternal trace element status, including manganese, prohibits a final conclusion and more research in this regard is needed.

Regarding maternal and child complications, in agreement with previous studies [28,41], no significant association between zinc and selenium concentrations and various measures of adverse pregnancy outcomes were found. However, some other studies found that maternal zinc deficiency during pregnancy elevates the risks of foetal growth restriction [11]. Similarly, low serum selenium status could contribute to low birth weight [42], small for gestational age infants [43], and the risk of developing pre-eclampsia and pregnancy-induced hypertension [44].

Concurrently, we observed that higher manganese concentrations in pregnant women were associated with an increased odds ratio of maternal complications. However, the results with manganese were only an association and should be treated cautiously, as correlation does not necessarily mean causality. More studies are definitely necessary to evaluate the effects of manganese status on maternal and neonatal outcomes.

Because the knowledge of the effects of manganese exposure during pregnancy remain limited, few previous studies have reported an inverted U-shaped relation between maternal manganese levels and birth outcomes [18]. However, other studies found that increased manganese concentration during pregnancy might be a potential risk factor for developing pregnancy hypertension [19], intrauterine growth restriction [45], and preeclampsia [46]. Manganese, as an essential component of the enzyme Mn-superoxide dismutase (MnSOD), protects the energy-generating mitochondria from oxidative stress [14], whereas manganese toxicity could be due to manganese-induced cellular free radical damage, reactive oxygen species, toxic metabolites, alteration of mitochondrial function, and ATP production [47]. During pregnancy, the increase in manganese levels might be related to increased erythropoiesis, intestinal absorption, or tissue manganese mobilization [48]. In particular, females from Asia have been reported to have higher manganese levels than other ethnic groups [49].

By applying an FFQ, we found that women from Pakistan principally consumed grains (whole wheat), which is the main staple food consumed in the country. In contrast, not merely meat and nuts, but fish consumption was also low in our sample. With few exceptions, we did not find significant correlations between the intake of different foods and the trace element status. Our data tend to confirm previous findings and showed the inverse correlation of dairy products and vegetables with selenium and manganese levels, respectively [50]. Moreover, we found an inverse relationship between the consumption of fruits and serum zinc concentration. Different dietary components including phytate content of foods have been shown to influence zinc bioavailability [36].

Although cereals are the major source of phytate, it is widely present in plant-based diets, nuts, vegetables, and in some fruits [51]. Additionally, we found a positive relationship between the consumption of whole grains and manganese concentration. Whole grains have been considered as a rich source of manganese in human diets [52], and our study participants frequently consumed this nutritional component. Manganese levels in drinking water vary depending on the geographical area and contamination [53]. Therefore, the WHO recommended manganese concentrations at 0.4 mg/l for safe drinking water and avoiding toxicity [54]. Likewise, selenium levels in soil generally are influenced by geographical location, seasonal changes, protein content, and food processing [55]. Therefore, an adequate selenium intake of at least 40 µg/day is suggested to support the maximal expression of the selenium enzymes [56].

### Strengths and Limitations

This study was probably the first Pakistani study to emphasize the status of selenium, zinc, and manganese during pregnancy and their relation to pregnancy complications and perinatal outcomes. However, our study had a few limitations. Different socio-demographic factors may affect maternal trace element status and foetal outcomes [57]. We are confident that we controlled for major covariates, however, as it is well known in observational studies, we cannot exclude that also other potential confounders might have affected the results, at least to some extent. Because the sample size for each maternal or child complication was relatively low, we therefore fused all maternal and child complications. This fusion could be regarded as another limitation. Moreover, women taking multivitamins during pregnancy were excluded from the study, however, information regarding multivitamins before conception was not recorded, which could also be considered as a potential limitation. However, it is known that women in Pakistan seldom use multivitamin supplements [58]. Moreover, FFQs consider the intake of foods over a longer period of time, and therefore the accuracy of reported food intake such as meal patterns, variable portion size, and some smaller portions of single food items were possibly not indicated. In addition, the FFQ did not distinguish between cooking methods or if the foods were fresh or frozen, which can be counted as a certain study limitation.

## 5. Conclusions

Our study showed lower serum selenium and zinc levels in pregnant women as compared to non-pregnant controls, whereas no difference in manganese concentration was found. Additionally, the results suggest that high maternal serum manganese concentrations may affect maternal complications.

Because adequacy of micronutrient intake and consequent blood concentration during pregnancy have also been stated to be affected by different environmental, cultural, and demographic variables [59], it is crucial to consider depleted nutrients from food intake and future nutritional interventions at the population level. Although several previous studies in different geographic regions have examined trace element concentrations in pregnant women, it is likely that no other study, except the present one, has emphasised the association of trace element status with maternal and child complications in Pakistan. Further studies are needed to clarify the role of food intake on the status of trace elements in pregnant women in the country to optimise the efforts regarding maternal nutrient intake, supplementation, or food fortification.

## Figures and Tables

**Table 1:** Characteristics of study participants (mean ± SD or *n* (%)).

		Control Group	Pregnant Women
Characteristic	Category/Unit	*n* = 40	*n* = 80
Age	Years	25 ± 4	26 ± 4
Marital status	Married	22 (55.0%)	80 (100.0%) *
	Not married	18 (45.0%)	0 (0.0%)
Education	No education	19 (47.5%)	45 (56.3%)
	Primary or high school education	21 (52.5%)	35 (43.7%)
Occupation	Housewife	22 (55.0%)	78 (97.5%) *
	Working	18 (45.0%)	2 (2.5%)
Family type	Single family	25 (62.5%)	21 (26.3%) *
	Joint family	15 (37.5%)	59 (73.8%)
Children	Number	1.7 ± 2.0	1.6 ± 2.1
Pregnancy complications	Yes	0 (0.0%)	26 (32.5%) ^N.A^
	No	0 (0.0%)	54 (67.5%)
Previous miscarriages	Yes	5 (12.5%)	27 (33.8%) *
Physical activity	Low	18 (45.0%)	14 (17.5%) *
	Moderate/high	22 (55.0%)	66 (82.5%)
Income	$/month	599 ± 692	183 ± 157 *
BMI ^a^	kg/m^2^	25.6 ± 4.0	27.0 ± 2.9 *

^a^ BMI (body mass index) was recorded at the time of delivery, * statistically significant (<0.05) compared to the control group, ^N.A^ not applicable

**Table 2:** Concentrations of selenium, zinc, and manganese in blood serum of pregnant women as compared to the control group.

Trace Element	Control Group (Mean ± SD) ^b^	Pregnant Group (Mean ± SD) ^b^	95% Confidence Interval of Difference	*p*-Value ^a^
Selenium (µmol/L)	2.76 ± 1.15	2.26 ± 1.09	[0.05; 0.97]	0.031
Zinc (µmol/L)	29.54 ± 7.62	21.86 ± 7.21	[4.63; 10.71]	<0.001
Manganese (log_10_ nmol/L)	1.38 ± 0.09	1.40 ± 0.09	[-0.05; 0.02]	0.365

**^a^** General linear model controlled for age, BMI, education, income, physical activity, and parity; ^b^ Adjusted for covariates.

**Table 3:** Concentrations of selenium, zinc, and manganese in blood serum of pregnant women (primipara vs. multipara).

Trace Element	Primipara (Mean ± SD) ^b^	Multipara (Mean ± SD) ^b^	95% Confidence Interval of Difference	*p*-Value ^a^
Selenium (µmol/L)	2.48 ± 1.18	2.25 ± 1.18	[−0.33; 0.79]	0.427
Zinc (µmol/L)	21.61 ± 8.03	22.48 ± 8.03	[−4.69; 2.95]	0.656
Manganese (log_10_ nmol/L)	1.43 ± 0.10	1.38 ± 0.10	[0.01; 0.10]	0.025

**^a^** General linear model controlled for age, BMI, education, income, physical activity, antenatal visits, and previous miscarriages; ^b^ Adjusted for covariates.

**Table 4:** Association of selenium, zinc, and manganese in blood serum of pregnant women with the risk of maternal and child complications ^a,b^.

	Odds Ratio	95 % Confidence Interval	*p*-Value
**Selenium**			
Maternal Complications	1.006	[0.605; 1.673]	0.981
Child Complications	0.728	[0.351; 1.509]	0.393
**Zinc**			
Maternal Complications	0.921	[0.842; 1.007]	0.071
Child Complications	0.997	[0.902; 1.102]	0.954
**log_10_-Manganese ^c^**			
Maternal Complications	3.175	[1.631; 6.181]	0.038
Child Complications	1.420	[−1.999; 4.820]	0.413

**^a^** Dependent variable was maternal or child complications (yes/no); ^b^ controlled for age, parity, education, income, physical activity, and antenatal visits; **^c^** manganese was logarithmically transformed.

**Table 5:** Stepwise multiple linear regression analysing the association of the intake of different foods and beverages on the status of selenium, zinc, and manganese ^a,b^.

Trace Element	Food Item	β-Coefficient	*p*-Value
**Selenium**			
	Dairy Products	−0.057	0.025
**Zinc**			
	Fruits	−0.462	0.030
	Cold Drinks	0.969	0.002
**Manganese (log_10_)**			
	Grains	0.508	0.002
	Vegetables	−1.045	0.001
	Dairy Products	−0.638	<0.001
	Sweets	1.423	0.013

^a^ Only significant results are presented; ^b^ controlled for age, BMI, income, physical activity, and marital status.
